# Wind speed acceleration around a single low solid roughness in atmospheric boundary layer

**DOI:** 10.1038/s41598-019-48574-7

**Published:** 2019-08-19

**Authors:** Lin-Tao Fu, Qing Fan, Zong-Liu Huang

**Affiliations:** 1 School of Mechanical Engineering, Chengdu University, Chengdu, 610106 China; 2 Zhuhai Campus of Zunyi Medical University, Zhuhai, 519041 China; 3 Key Laboratory of Fluid and Power Machinery, Ministry of Education, Xihua University, Chengdu, 610039 China

**Keywords:** Atmospheric dynamics, Environmental impact, Climate and Earth system modelling, Atmospheric dynamics, Geomorphology

## Abstract

Air flow around vegetation is crucial for particle transport (e.g., dust grains, seeds and pollens) in atmospheric boundary layer. However, wind acceleration around vegetation is still not well understood. In this work, air flow around a single low solid roughness element (representing a dense shrub patch or clump) in atmospheric boundary layer was numerically investigated, with emphasizing wind acceleration zone located at the two lateral sides. The maximum value of dimensionless horizontal wind speed as well as its location of occurrence and the geometrical morphology and area of wind acceleration zone were systematically studied. It reveals that they could alter significantly with the change of roughness basal shape. The maximum value of dimensionless resultant horizontal speed decreases monotonously with observation height, while the area of wind acceleration zone shows a non-linear response to observation height. The dependence of the maximum speed location on observation height is generally weak, but may vary with roughness basal shape. These findings could well explain the disagreement among previous field observations. We hope that these findings could be helpful to improve our understanding of aeolian transport in sparsely vegetated land in arid and semi-arid region, and wind dispersals of seeds and pollens from shrub vegetation.

## Introduction

Vegetation plays very important role in planetary ecosphere. The existence and the change of vegetation may significantly affect land surface processes and the evolution of terrestrial climate^1–3^. In atmospheric boundary layer, the interaction between wind and vegetation could have direct or indirect impacts on vegetation. Wind may compel trees to alter their architecture or even break trees^4,5^. Seed dispersal by wind is one of main driving factors for temporal and spatial evolution of vegetation communities^6,7^. More importantly, through exchanging momentum with wind field, vegetation could reduce wind speed near ground and consequently decrease the risk of surface wind erosion^1,8,9^. Therefore, many studies have been focused on the shear stress partitioning and surface aerodynamic parameters in the cases of rigid roughness^10–13^ and flexible roughness (plant model or real plant)^14–16^ to improve the prediction of aeolian flux in the presence of vegetation. In practice, owing to the sheltering effect of vegetation, Straw Checkerboard Barriers have also been employed to control or slow down regional land desertification, through considering the characteristics of turbulent flow, particle motion, and internal erosion form within Straw Checkerboard Barriers^17^.

However, uncertainties still exist in estimations of the magnitude of dust events^18^ or of the exact location of dust sources^19^ in dust forecast models, which possibly originates from the shear stress partitioning model^20^. Recent study revealed the uncertainty may increase for very low vegetation density near threshold wind speed^21^. This is because vegetation could not only lead to local wind reduction on the leeside^8,22–25^, but also result in wind acceleration at the two lateral sides^23–26^. In fact, the lateral wind acceleration may contribute markedly to fluid-entrained dust from surface for very low vegetation density near threshold wind speed^21^. In arid and semi-arid regions, particularly on desert edge, the lateral density of shrub (dominant vegetation type) or shrub cover is typically very low^23^. In such case, shrub vegetation stands as a single patch or clump (called as vegetation element hereafter) with various shapes^23,27–29^. And, the distance among shrub elements is so large (larger than 15~20 times of shrub height) that the interaction among elements could be ignored^23^. Therefore, theoretical, experimental and numerical methods were employed to investigate wind speed around vegetation element^8,23–25,30–32^, particularly for a single shrub element standing at sparsely vegetated land surface in arid and semi-arid regions^23–26^.

Most studies about wind speed around vegetation focused on wind speed reduction on the leeside because of the role of vegetation in protecting from wind erosion. Raupach^8^, Okin^22^ and Leenders *et al*.^24^ proposed triangular, rectangular and semi-elliptical shapes for “protecting region” (in which wind erosion is reduced or completely disappears) formed by vegetation element, respectively. Raupach^8^ ideally supposed that surface wind shear stress within protecting region equals zero, which means there is no erosion. Okin^22^ suggested that the shear stress within protecting region was not a fixed value but evolved gradually. Based on experimental data from porous windbreaks^33^, he proposed empirical relationship between shear stress and leeward distance (scaled by vegetation height) within protecting region. Leenders *et al*.^24^ and Mayaud *et al*.^25^ also confirmed the gradual variation of shear stress with downward distance. Based on field observation data, they further developed new empirical relationships which include both vegetation porosity and height. Besides, Cheng *et al*.^30,31^ and Liu *et al*.^32^ conducted both wind tunnel experiments and CFD numerical simulations to study the influences of vegetation architecture on wind speed, and proposed an improved empirical relationship which includes vegetation porosity, height and width.

In contrast, the study about wind acceleration at the lateral sides of shrub vegetation is paid less attention, although studies about wind acceleration around other small roughness^34,35^ and trees^31^ have been conducted. Ash and Wasson^26^ measured wind speeds at the lateral sides of dense shrub by hand-held cup anemometer in field, and they found that the maximum degree of acceleration of wind speed at the side of shrub could be up to 130% (the ratio of measured wind speed versus referring wind speed at a fixed height, called “maximum value of dimensionless resultant horizontal speed” hereafter). They drew out the morphology of wind acceleration zone qualitatively, but missed to describe it quantitatively. Leenders *et al*.^23,24^ proposed an elliptical shape of wind acceleration zone based on their field data. The windward and the lateral axes of the ellipse were, respectively, set to be 0.5 times of *T* (stream-wise thickness of shrub element) and 0.25 times of *W* (frontal width of shrub element). Within this elliptical wind acceleration zone, the mean of dimensionless horizontal speed is 1.06, with the maximum value of 1.12. Fu^21^ investigated the impact of wind acceleration on predicted dust release by using these parameters, and found that, in comparison to the area of wind acceleration zone, predicted dust release showed higher sensitivity to values of dimensionless wind speed. Moreover, Mayaud *et al*.^25^ showed very weak degree of wind acceleration among their field measurements.

In general, our understanding of wind acceleration at two the lateral sides of single vegetation element is far from enough. From a review of previous studies, there are two questions required to be resolved. The first question is how wind acceleration changes with observation height, and, the second one is whether vegetation shape influences wind acceleration or not. Due to the limitation of the size and the amount of instruments, the resolution of meshing grids for measurements (i.e., the space between neighboring measuring points and the distance from measuring points to vegetation in previous studies mentioned above) isn’t high enough. It is thus difficult to obtain more detailed information about wind speed around vegetation from direct field measurements. Therefore, this work aims to solve these two questions partially through numerically modeling wind speed around a single dense shrub (represented by solid roughness of identical size) in atmospheric boundary layer under various conditions.

## Materials and Methods

### The governing equations

Atmospheric boundary layer is assumed to be neutral. The air flow is considered to be incompressible and Newtonian. The three dimensional RANS equations of mass conservation and momentum conservation are thus given by Eqs (1) and (2)^36^, respectively, where, *t*, *x*_*i*_, ${\bar{u}}_{i}$, $\bar{p}$, $\overline{{u^{\prime} }_{i}{u^{\prime} }_{j}}$ and *S*_*i*_ (*i*, *j* = 1, 2, 3) are the time, the Cartesian coordinates, the mean velocity components, the mean pressure, the Reynolds stresses and the source term, respectively. *ρ* is the air density and taken as 1.225 kg/m^3^.*η*_*a*_ is the air dynamic viscosity and taken as 1.78 × 10^−5^ kg/(ms). *ν* is the air kinematic viscosity and defined as *ν* = *η*_*a*_/*ρ*. The source term *S*_*i*_ equals zero.

1 $$\frac{\partial {\bar{u}}_{i}}{\partial {x}_{i}}=0$$

2 $$\frac{\partial {\bar{u}}_{i}}{\partial t}+{\bar{u}}_{j}\frac{\partial {\bar{u}}_{i}}{\partial {x}_{j}}=-\,\frac{1}{\rho }\frac{\partial \bar{p}}{\partial {x}_{i}}+\frac{\partial }{\partial {x}_{j}}(\nu (\frac{\partial {\bar{u}}_{i}}{\partial {x}_{j}}+\frac{\partial {\bar{u}}_{j}}{\partial {x}_{i}})-\overline{{u^{\prime} }_{i}{u^{\prime} }_{j}})+{S}_{i}$$

### Turbulence model

Turbulence is simulated by standard *k*-*ε* model^37^, and standard wall functions are employed^38^. The Reynolds stresses are modeled by Eq. (3), where the kinematic turbulent viscosity *ν*_*t*_ is calculated as *ν*_*t*_ = *C*_*μ*_*k*^2^/*ε*. Here, C_μ_ = 0.09, *k* and *ε* are the turbulent kinetic energy and the dissipation rate, respectively. The kinetic energy and the dissipation rate are closed by Eqs (4) and (5), respectively, where *G*_*k*_ is the turbulent kinetic energy production, caused by the product of the Reynolds stress and the rate of strain. Other constants for turbulence model are *C*_1*ε*_ = 1.44, *C*_2*ε*_ = 1.92, *σ*_*k*_ = 1.0 and *σ*_*ε*_ = 1.3.

3 $$-\overline{{u^{\prime} }_{i}{u^{\prime} }_{j}}={\nu }_{t}(\frac{\partial {\bar{u}}_{i}}{\partial {x}_{j}}+\frac{\partial {\bar{u}}_{j}}{\partial {x}_{i}})-\frac{2}{3}k{\delta }_{ij}$$

4 $$\frac{\partial k}{\partial t}+\frac{\partial ({\bar{u}}_{i}k)}{\partial {x}_{i}}=\frac{\partial }{\partial {x}_{j}}((\nu +\frac{{\nu }_{t}}{{\sigma }_{k}})\frac{\partial k}{\partial {x}_{j}})+\frac{1}{{\rm{\rho }}}{G}_{k}-\varepsilon $$

5 $$\frac{\partial \varepsilon }{\partial t}+\frac{\partial ({\bar{u}}_{i}\varepsilon )}{\partial {x}_{i}}=\frac{\partial }{\partial {x}_{j}}((\nu +\frac{{\nu }_{t}}{{\sigma }_{\varepsilon }})\frac{\partial \varepsilon }{\partial {x}_{j}})+\frac{1}{{\rm{\rho }}}{C}_{1\varepsilon }\frac{\varepsilon }{k}{G}_{k}-{C}_{2\varepsilon }\frac{{\varepsilon }^{2}}{k}$$

### Simulation domain and vegetation model

**Figure 1:**
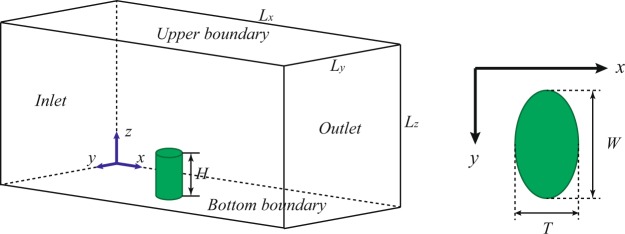
Diagrammatic sketch of simulation domain as well as vegetation model. Right panel: top view of roughness element. *H*, *W* and *T* are vegetation height, frontal width and stream-wise thickness, respectively.

All simulations are performed with commercial CFD software ANSYS FLUENT (package version 15.0). Simulation domain and vegetation model are shown in Fig. 1, where, *x*, *y* and *z* suggest stream-wise, lateral, and vertical directions, respectively. *L*_*x*_, *L*_*y*_ and *L*_*z*_ represent the length, width and height of simulation domain, respectively. As mentioned above, shrub is considered to be dense enough (namely porosity equals zero). So, shrub could be simply modeled as solid roughness. In previous studies^21,22^, shrub element was usually simplified as circular cylinder. However, actual shrub shape should be diverse^23,27–29^. And, wind force may compel shrub to alter its shape depending on the flexibility of branches. Therefore, shrub element is assumed to elliptical cylinder which is parameterized by vegetation height *H*, frontal width *W* and stream-wise thickness *T* (Fig. 1). In order to avoid the scale effect of model size on simulation results^39^, the height *H* and the width *W* of vegetation model are set to be 0.5 m and 1.0 m, respectively, based on field observations^23,25^. Thus, the length (*L*_*x*_), the width (*L*_*y*_) and the height (*L*_*z*_) of simulation domain are set to be 50 m, 10 m and 10 m, respectively. Vegetation model is fixed at the distance 20 m downwind from inlet. Structured multi-block mesh is applied, and grids are made finer near both ground surface and vegetation element (Details see Fig. S1). The first layer (the minimum grid) near the wall has the height of 0.02 m, and the total number of grids is around 6 × 10^5^. In the cases of modeling flow around vegetation roughness element, the flow is assumed to be steady. The residual criteria of the simulations are set to be 1 × 10^−6^.

### Boundary conditions

6a $$U(z)=\frac{{u}_{\ast }}{\kappa }In(\frac{z}{{z}_{0}})$$

6b $${z}_{0}={k}_{s}exp(\,-\,\kappa {B}_{rek})$$

6c $$\{\begin{array}{rcl}{{\rm{B}}}_{{\rm{rek}}} & = & 8.5+(2.5{{\rm{lnRe}}}_{{\rm{k}}}-3)\exp [-0.11{({{\rm{lnRe}}}_{{\rm{k}}})}^{2.5}],\,{{\rm{Re}}}_{{\rm{k}}} > 1\,\\ {{\rm{B}}}_{{\rm{rek}}} & = & 8.5+(2.5{{\rm{lnRe}}}_{{\rm{k}}}-3),\,{{\rm{Re}}}_{{\rm{k}}}\le 1\,\end{array}$$

At the inlet, the logarithmic wind profile (Eq. 6a) is imposed. *κ* is the von Karman’s constant and taken as 0.41, *z*_0_ the aerodynamic surface roughness, and u_*_ wind shear speed. *z*_0_ is calculated by Eq. 6b^40^, where *k*_*s*_ is the equivalent Nikuradse roughness and *B*_*rek*_ is a function of the roughness Reynolds number (defined as *Re*_*k*_ = *k*_*s*_u_*_/*ν*) (Eq. 6c). A pressure outlet boundary is applied at the outlet. At the inlet *k* and *ε* are defined as ${{\rm{u}}}_{\ast }^{2}/{{\rm{C}}}_{{\rm{\mu }}}$ and ${{\rm{u}}}_{\ast }^{3}/({\rm{\kappa }}z)$, respectively. The turbulent kinetic energy (*k*) and turbulent dissipation rate (*ε*) at the outlet are set to be identical with those at the inlet. A symmetry boundary is imposed at the two lateral sides of simulation domain^41^. A non-slip boundary condition is applied to solid-fluid interfaces (at bottom boundary and solid roughness)^42^. In this work, the land surface is supposed to be crusted soils consisting of mixed grain size with average diameter *d* = 0.25 mm^43^. Thereby, *k*_*s*_ is determined as 2*d*^44^. In ANSYS FLUENT, the value of roughness constant *C*_*s*_ is also required apart from *k*_*s*_. A recommended constant value 0.5 is not used here because *C*_*s*_ will change with wind shear speed. Instead, *C*_*s*_ is reversely calculated by combining Eq. 6b and formula *k*_*s*_ = 9.793*z*_0_/*C*_*s*_^45^. The variation of *C*_*s*_ with shear speed could be found in Fig. S2. Because the height of simulation domain is only 10 m (much smaller than the thickness of atmospheric boundary layer^41^), the shear speed at upper boundary is set to be u_*_ (the same value at inlet) as previous study did^46^. Consequently, turbulent kinetic energy and turbulent dissipation rate wouldn’t change along the length of upper boundary^47^.

### Verification of numerical method

**Figure 2:**
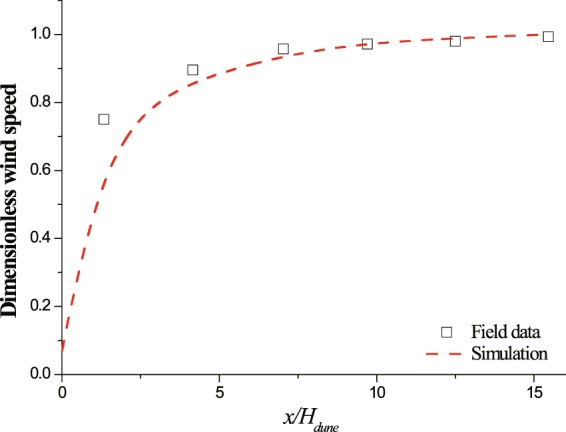
Comparison of leeward dimensionless wind speed between field observation and simulation.

With those settings above, the vertical profiles of time-averaged stream-wise wind speed at different locations downwind from the inlet, in the case of without including vegetation model, were tested (see Fig. S3). It could be found that all vertical profiles at different locations almost coincide. The relative variations of wind speed at every height are smaller than 1%. Our settings are thus confirmed to be reasonable and applicable. To further test our numerical method, the measured wind recovery in the lee of an un-vegetated nebkha dune^48^ was compared with our simulated results. The nebkha dune has a height (*H*_*dune*_) of 1.16 m (under un-vegetated condition) and a basal diameter of 4.3 m. The reference wind speed, which was measured at 0.4*H*_*dune*_, suggests an incoming shear speed of u_*_ = 0.45 m/s. Field measurements suggested weak influence of sand motion on leeward wind speed recovery within the range of wind speed from 1.3 to 12.0 m/s at the height of 1.16 m^48^. The simulation of wind flow around the dune model here doesn’t include the feedback of sand movements for convenience. The total calculation time is 15 s. The fluid time step is 0.001 s. Considering the complexity of rough surface condition in field, the aerodynamic surface roughness *z*_0_ here is taken as 0.0005 m^49^. The variation of simulated dimensionless wind speed (the ratio of the wind speed at a stream-wise location versus the wind speed at the reference location at the same observing height) in the lee of the dune is shown in Fig. 2. It could be found that our simulated results agree well with measurements, apart from the nearest measuring location (relative to the lee of the dune). The discrepancy at the nearest measuring location may be caused by multiple factors. But, the main reason might be the employment of ideal 3D dune shape instead of real dune shape as previous study did^49^. Nevertheless, the comparison between simulated results and experimental data indicates that our numerical method could be capable of reproducing the change of leeward wind speed around a low solid roughness element in atmospheric boundary layer.

### Other settings

In order to investigate the influences of incoming wind speed, four wind shear speeds (u_*_ = 0.25, 0.30, 0.40 and 0.50 m/s) are employed. In all simulations, vegetation height is fixed as *H* = 0.5 m. The shape of shrub element is thus parameterized by the ratio of thickness versus width, i.e., *T/W*. For studying the influences of shrub shape, seven values of *T/W* (*T/W* = 0.2, 0.5, 0.8, 1.0, 1.2, 1.5 and 2.0) are employed. Besides, seven observation heights (*z/H* = 0.05, 0.1, 0.3, 0.5, 0.7, 0.9 and 1.0) are selected to explore the vertical variations of wind acceleration. In the following, *U(z)*_*xy*_ represents the resultant horizontal speed at location (x, y) in the horizontal plane of height z. *U(z)*_*xy,0*_ and *U(z)*_*xy,max*_ are the speed at the inlet and the maximum speed in the horizontal plane of height z, respectively. *U(z)*_*xy*_/*U(z)*_*xy,0*_ and *U(z)*_*xy,max*_/*U(z)*_*xy,0*_ are the dimensionless wind speed (value larger than 1 suggests wind acceleration) and the maximum dimensionless speed, respectively. The location of the maximum dimensionless speed is jointly determined by the distance *r*_*umax*_ (the distance from the location where the maximum speed occurs to the center of vegetation element) and the azimuth angle *Θ*_*umax*_. A schematic description of *r*_*umax*_ and *Θ*_*umax*_could be found in Fig. S4. During calculations of the area of wind acceleration zone, only the region in which dimensionless wind speed is larger than 1.02 is collected in view of possible numerical error. *A*_0_ and *A*_*in*_ represent the basal area of elliptical cylinder and the area of wind acceleration zone, respectively. Finally, the fluid-entrained dust *F*_*vt*_ (μg/s) at fluid threshold is estimated as ${F}_{vt}={A}_{in}9.88{u}_{\ast }^{10}(1-{u}_{\ast ft}/{u}_{\ast })\times {10}^{6}$^50^ if soil surface is assumed to be erodible. In previous studies^22,25^, shear speed was referred to the ratio of measured speed versus inlet speed at a fixed height. Here, shear speed used to calculate dust release is referred to the dimension horizontal speed at *z/H* = 0.05.The threshold shear speed u_*ft_ could be selected as 0.3 m/s^21^ if the average diameter of bed equals 0.25 mm as mentioned above.

## Results

### Geometrical morphology of wind acceleration zone in horizontal plane

**Figure 3:**
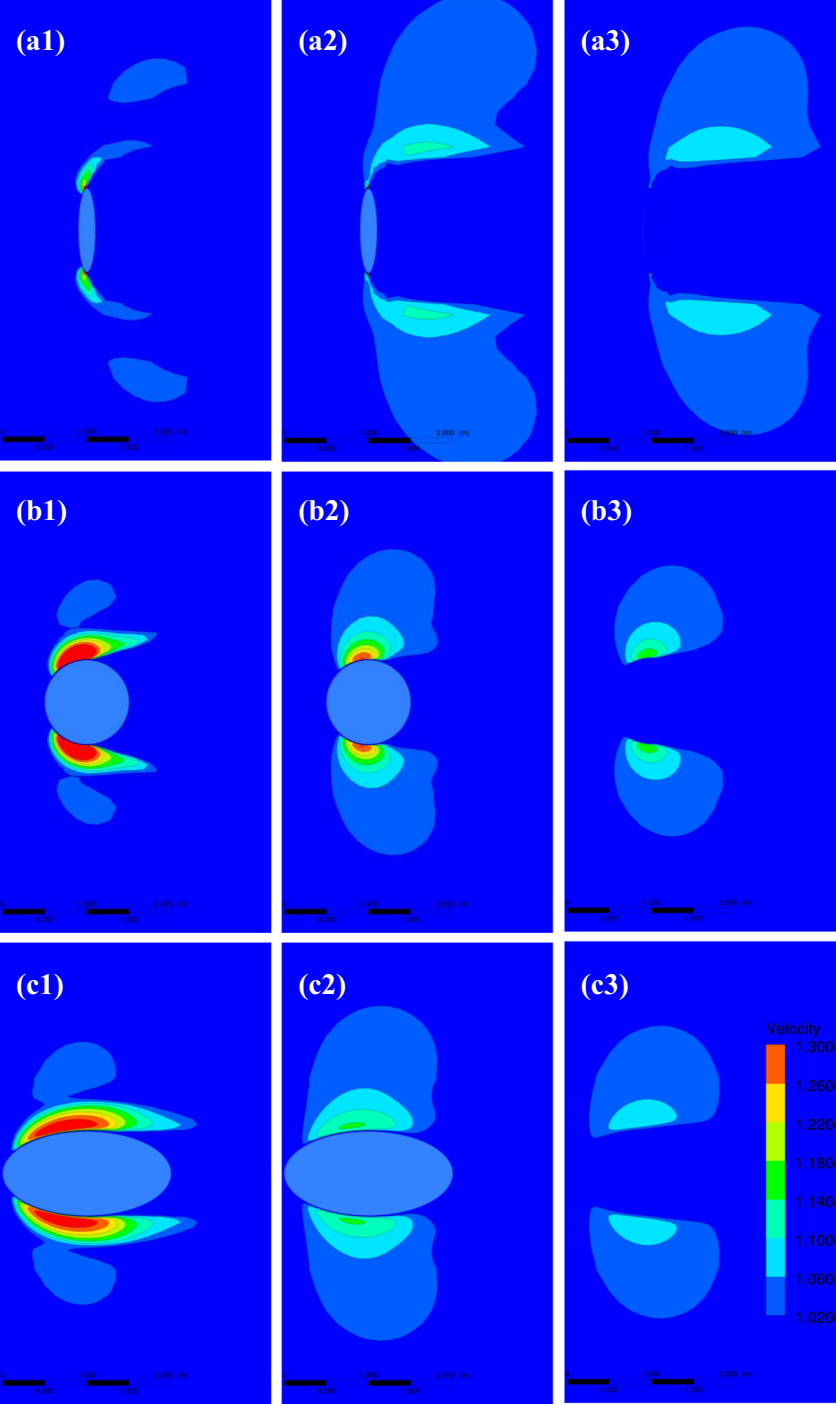
The vertical variations of wind acceleration zone at u_*_ = 0.5 m/s for different roughness shapes. The top three panels (a), middle three panels (b) and bottom three panels (c) correspond to *T/W* = 0.2, 1.0 and 2.0, respectively. Numbers 1, 2 and 3 correspond to *z/H* = 0.05, 0.5 and 1.0, respectively.

This subsection mainly aims to show how wind acceleration zone varies qualitatively. All contour maps in this section are plotted by dimensionless wind speed. The lowest value of dimensionless wind speed is 1.02 and the interval of dimensionless wind speed is 0.04. Figure 3 shows the vertical variations of wind acceleration zone at u_*_ = 0.5 m/s. It is clear that wind acceleration zone changes with observation height significantly. With the increase of observation height, wind acceleration zone alters from dispersed distribution to continuous distribution, and the morphology of the zone becomes regular gradually. The area of wind acceleration zone increases at first but decreases then with *z/H*. Generally, wind speed gradually decreases with the increase of the distance from a location to the center of vegetation element. The maximum value of dimensionless resultant horizontal speed shows a trend of monotonous decrease with observation height. The change of roughness shape wouldn’t alter the two variation laws of wind speed mentioned above. However, roughness shape could affect the area of wind acceleration zone. At *z/H* = 0.05 (Fig. 3a1,b1,c1), the area increases with the increase of *T/W*. But, at *z/H* = 0.5 (Fig. 3a2,b2,c2) and *z/H* = 1.0 (Fig. 3a3,b3,c3), the area decreases at first but increases then with the increase of *T/W*. Moreover, modeling results suggest that the effects of shear speed on wind acceleration zone could be ignored (See details in Fig. S5 and Table S1).

### Maximum horizontal speed and its location

**Figure 4:**
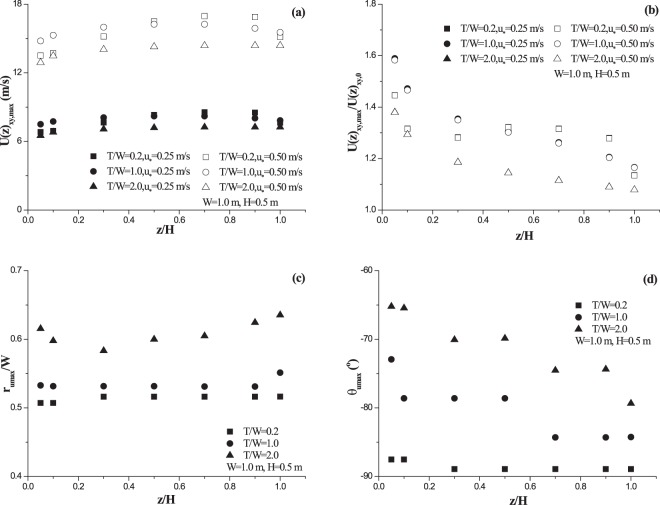
Vertical variations of maximum horizontal speed (**a**), maximum dimensionless horizontal speed (**b**), the distance from the location of the maximum speed to vegetation center (**c**), and the azimuth angle (**d**).

**Figure 5:**
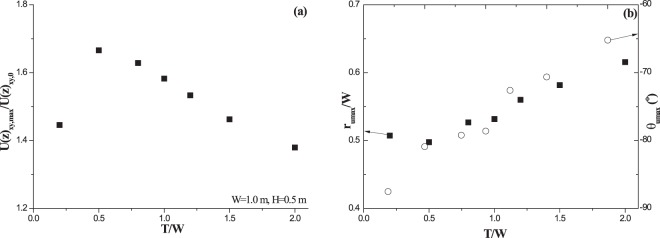
Maximum value of dimensionless horizontal wind speed (**a**) and its location (**b**) versus roughness shape *T/W* at *z/H* = 0.05.

It reveals that the maximum resultant horizontal speed in wind acceleration zone must increase with incoming wind speed at a fixed height (Fig. 4a). Figure 4a also shows that, within roughness height, the maximum resultant horizontal speed increases at first but then slowly decreases with observation height. This variation trend is independent on both incoming wind speed and roughness shape. In contrast, the maximum value of dimensionless resultant horizontal speed shows a distinct variation trend with observation height (Fig. 4b). It shows that incoming wind speed hardly affects the vertical variation of the maximum dimensionless speed. Nevertheless, the dimensionless speed shows high sensitivity to observation height. At *T/W* = 0.2, the maximum dimensionless horizontal speed changes with observation height in a complex way (decreasing firstly, increasing then, and decreasing finally). But, at *T/W* = 1.0 and *T/W* = 2.0, the dimensionless speed decreases with observation height monotonously. Figure 4c shows the variation of the distance from the occurrence location of the maximum speed to the center of vegetation element with observation height. It suggests that this distance could be almost independent on observation height, even though a small increase exists. Quantitative analysis reveals that the maximum relative error to averaged value (0.609) among all observation heights is less than 5% at *T/W* = 0.2 (In which the most significant fluctuation happens). It also reveals that the distance increases with the increase of *T/W* (averaged dimensionless distances are 0.514, 0.534 and 0.609). The sensitivity of azimuth angle to observation height seems to be dependent on roughness shape (Fig. 4d). For a fixed height, the azimuth angle (minus sign suggests its direction is opposite to flow direction) increases with *T/W*. The variation gradient of azimuth angle with height also increases with *T/W*. For example, at *T/W* = 0.2, the azimuth angle hardly change with observation height; but, at *T/W* = 2.0, the differential between the maximum value and the minimum value of azimuth angle could reach 14°. Furthermore, the effects of roughness shape on both the maximum value of dimensionless resultant horizontal speed and its location at *z/H* = 0.05 are quantitatively analyzed (Fig. 5). Figure 5a reveals that the maximum dimensionless resultant horizontal speed near surface increases at first but decreases then with *T/W*. Curve fitting by the least square method indicates that the formula, $U{(z)}_{xy,max}/U{(z)}_{xy,0}=1.367+0.305\,exp[\,-\,exp(-\zeta )-\zeta +1],\,\zeta =(T/W-0.655)/0.372$ (R^2^ > 0.93), could well describe the variation. However, the distance *r*_*umax*_ and the azimuth angle *Θ*_*umax*_ are found to change with *T/W* linearly (Fig. 5b). The change of *r*_*umax*_ could be well expressed by formula *r*_*umax*_/*W* = 0.476 + 0.067*T*/*W* (R^2^ > 0.92). The azimuth angle *Θ*_*umax*_ could be well written by formula *Θ*_*umax*_ = −88.7 + 12.0*T*/*W* (R^2^ > 0.95).

### Area of wind acceleration zone and induced dust emission

**Figure 6:**
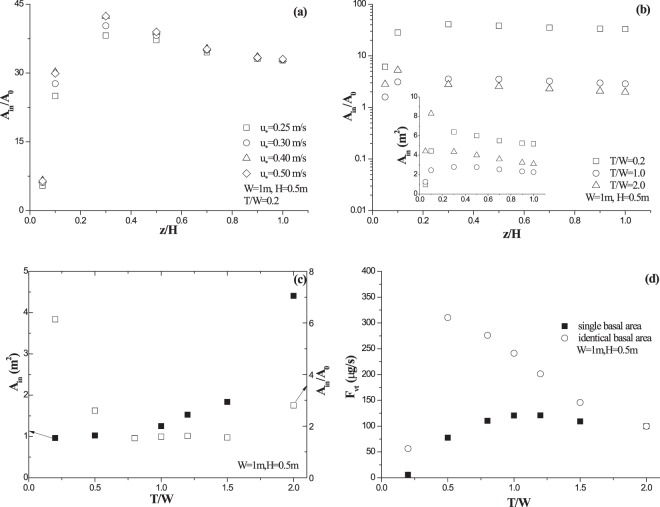
Variations of wind acceleration zone as well as predicted dust flux. Panels (a) and (b): area of wind acceleration zone *A*_*in*_ and dimensionless area *A*_*in*_/*A*_0_ versus observation height; panels (c): *A*_*in*_ and *A*_*in*_/*A*_0_ at *z/H* = 0.05 versus roughness shape *T/W*; panels (d): predicted dust flux *F*_*vt*_ versus roughness shape *T/W*.

Figure 6a shows the vertical variation of dimensionless area (*A*_*in*_/*A*_*0*_) at *T/W* = 0.2 for different incoming wind speeds. Due to the limited impact of incoming wind speed (Fig. S5 and Table S1), the area of wind acceleration zone for a fixed *T/W* in the following is calculated as the mean value of four incoming wind speeds. As pointed out in Section 3.1, dimensionless area increases at first and decreases then with observation height, and the maximum value of dimensionless area occurs around *z/H* = 0.3. Figure 6b shows that both the area of wind acceleration zone *A*_*in*_ and the dimensionless area *A*_*in*_/*A*_*0*_ at *T/W* = 0.2 for *z/H* = 1.0 are the largest among three cases of *T/W*. For *z/H* = 0.05, although *A*_*in*_ is the smallest at *T/W* = 0.2, however, *A*_*in*_/*A*_*0*_ at *T/W* = 0.2 is still the largest among three cases of *T/W*. This seems to indicate that the efficiency per basal area of roughness element in inducing wind acceleration area at *T/W* = 0.2 is the highest. Figure 6c shows the variations of both *A*_*in*_ and *A*_*in*_/*A*_0_ with *T/W* near surface (*z/H* = 0.05). It reveals that, *A*_*in*_ grows monotonously with *T/W*, while *A*_*in*_/*A*_0_ decreases at first and increases then with *T/W*. Data analysis suggests that *A*_*in*_ increases with *T/W* exponentially, namely, *A*_*in*_ = 0.966 + 0.021exp(2.56*T*/*W*) (R^2^ > 0.98); while, the variation of *A*_*in*_/*A*_0_ could be roughly expressed by a parabolic curve, namely, *A*_in_/*A*_0_ = 7.305−9.330*T*/*W* + 3.585(*T*/*W*)^2^ (R^2^ > 0.85). Although all simulation results are based on the assumption that soil surface is non-erodible, findings here could be applicable at fluid threshold wind. This is because the eroded dust flux at this threshold is so low that the feedback of dust flux to air flow could be ignored. Therefore, dust release around roughness element for different shapes is predicted (Fig. 6d). Two cases are considered here. In the first case, dust flux is predicted by using the area of wind acceleration zone of a single roughness element (solid square scatters in Fig. 6d). In the second case, dust flux is predicted by using identical area of wind acceleration zone (solid circle scatters in Fig. 6d). For convenience, this “identical” area is set to be the same as the area of wind acceleration zone of a single roughness element at *T/W* = 2.0. Figure 6d shows that the dust flux at threshold wind increases at first and decreases then with the increase of *T/W* for two cases. However, the maximum flux occurs at *T/W* = 1.0 for a single element (the first case), while it occurs at *T/W* = 0.5 for identical area (the second case). This difference is caused by the fact that dust flux is determined by both erodible area and wind speed. Although the area of wind acceleration zone increases with *T/W* (Fig. 6c), however, the degree of wind acceleration increases at first but decreases then with *T/W* (Fig. 5a).

## Discussion and Conclusion

It is a classic issue that fluid flows around a cylinder under different conditions. Field measurements^23–26^, numerical simulations^30,34^ and wind tunnel measurements^14,30,31^ have detected the wind acceleration around vegetation element. However, a systematic study on wind acceleration zone around vegetation element in atmospheric boundary layer is still missing. In the following, based on our simulation results, three aspects (the maximum value of dimensionless resultant horizontal speed, the area of wind acceleration zone and erosion flux in wind acceleration zone) are simply discussed.

In the study of Ash and Wasson^26^, reported maximum value of dimensionless resultant horizontal speed could be up to 1.3, which is much higher than the value 1.12 given by Leenders *et al*.^23,24^ and the finding from Mayaud *et al*.^25^. The reported distance *r*_*umax*_/*W* and the azimuth angle *Θ*_*umax*_ by Ash and Wasson^26^ are roughly 0.7 (about ranging from 0.6~0.8) and −80°, respectively. The distance *r*_*umax*_/*W* and the azimuth angle *Θ*_*umax*_ by Leenders *et al*.^24^ are 0.75 and −90 °, respectively. These disagreements among field observations could be explained from three points on the basis of our results. The first point is the difference in vegetation architecture. The vegetation element in Ash and Wasson^26^ is dense shrub, which suggests the porosity could be roughly zero. In the studies of Leenders *et al*.^23,24^ and Mayaud *et al*.^25^, vegetation is porous (porosity ranging from 0.3 to 0.7). In our simulations, vegetation is modeled as solid roughness, which makes our results close to the study of Ash and Wasson^26^. The second one is the difference in observation height. Our reported maximum value of dimensionless resultant horizontal speed near surface locates from 1.38 to 1.67 when *T/W* ranges from 0.2 to 2.0 (Fig. 5a), which is a little higher than Ash and Wasson^26^ but much larger than both Leenders *et al*.^23,24^ and Mayaud *et al*.^25^. This is because the maximum value of dimensionless resultant horizontal speed at a fixed location (x, y) decreases along with observation height (Fig. 4b). In the study of Leenders *et al*.^23,24^, observation heights (*z/H*) for two kinds of shrub are 0.24, 0.34, 0.45 and 0.67, respectively. In the study of Mayaud *et al*.^25^, observation heights (*z/H*) concentrates between 0.4~0.6, apart from one height *z/H* = 0.24. Hence, the combination of observation height and architecture may be the main reason why their reported values are much lower. Although we couldn’t make sure the actual observation height in Ash and Wasson^26^, however, we might infer their measuring height (*z/H*) locating within 0.1~0.3 (referring to Fig. 4b). The last one is the difference in vegetation shape. Our results show vegetation shape could affect the maximum value of dimensionless resultant horizontal speed remarkably (Figs 4 and 5a). Vegetation element is usually considered to be circular cylinder in theoretical studies, but actual shape may be diverse. Because of the flexibility of branches, vegetation architecture (or shape) may be compelled (most likely) from large *T/W* to small *T/W* by wind force^51^. Difference in shrub types and growth stages could result in different flexibilities, which thus increases the diversity or the uncertainty in final deformed shape of vegetation. It should be reminded that all results shown here are based on the assumption that wind direction is parallel or perpendicular to the major axis of elliptical roughness basal shape. The incident angle of flow (or the orientation of roughness element) could affect the flow field around roughness^52,53^. Therefore, the influence of flow incident angle on wind acceleration zone should be focused in the future once *T/W* doesn’t equal 1. Besides, although the location where the maximum speed occurs would be affected by observation height (Fig. 4c,d) and vegetation shape (Fig. 5b), however, the effect of vegetation porosity on the location is still unclear. In view of that published azimuth angles (*Θ*_*umax*_) are well located within the range of our results and only the published distances (*r*_*umax*_/*W*) are larger than our results, we infer that the spatial distribution of measuring points is the main reason for the disagreement in the location between measurements and our results.

Our results indicate that the morphology of wind acceleration zone change with both observation height and vegetation shape (Figs 2 and 3). However, the morphology could not describe by familiar and simple geometrical shape directly, which results in difficulty in applications, for example, the calculation of the area of wind acceleration zero. Leenders *et al*.^24^ proposed an elliptical shape of wind acceleration zone, which makes it easy to calculate the acceleration area by using semi-major axis and semi-minor axis. However, the proposal didn’t include the vertical variation of wind acceleration zone, which is inconsistent with our results (Fig. 6). The proposal of Leenders *et al*.^24^ suggests that the ratio of total wind acceleration area versus the basal area of vegetation element equals 1. Our results reveal that the ratio of total acceleration area versus basal area near surface is larger than 1.5 in all cases of *T/W*, and it varies with *T/W* in a nonlinear law (Fig. 6c). This difference might be caused by vegetation architecture. The vegetation model here is dense, while the vegetation in Leenders *et al*.^24^ is porous. Nevertheless, it is still unclear that how the porosity of vegetation element affects wind acceleration. Further research could therefore focus on quantifying the effect of porosity.

The measured maximum value of dimensionless resultant horizontal speed and the proposed area of wind acceleration zone in Leenders *et al*.^23,24^ are lower than our results. The estimated dust flux at threshold wind should thus be smaller than our results. In detail, if *W* = 1 m, the estimated dust flux for a single element is 1~2 orders of magnitude (17~98 times) higher than that predicted by the proposal of Leenders *et al*.^24^, when *T/W* ranges from 0.2 to 2.0. This suggests that the importance of vegetation architecture in the interaction between vegetation and air flow in atmospheric boundary layer. It also indicates that wind acceleration induced by dense vegetation could be an important reason for uncertainty in dust forecast model^21^. However, the factors that control the threshold wind speed of dust release are diverse^1,2^. For example, soil moisture is one of the most important factors for dust entrainment threshold^54–56^. Moisture should therefore be considered for estimating dust release in very sparsely vegetated land by applying our findings.

In conclusion, the flow field around a single low solid roughness element (representing dense shrub vegetation) in atmospheric boundary layer was numerically investigated, with emphasizing the wind acceleration zone located at two the lateral sides. The soil surface was assumed to be non-erodible and consisting of mixed grain size. The maximum value of dimensionless resultant horizontal speed, the morphology and the area of wind acceleration zone were systematically studied. The effects of observation height and vegetation shape (*T/W*) were investigated. The maximum value of dimensionless resultant horizontal speed decreases monotonously with observation height, and the maximum dimensionless speed near surface (*z/H* = 0.05) increases at first but decreases then with the increase of *T/W*. For a fixed *T/W*, the distance from occurrence location of maximum dimensionless speed to vegetation center is almost independent on observation height, but the azimuth angle alters with observation height (depending on *T/W*). The occurrence location (both the distance and the azimuth angle) of maximum dimensionless speed near surface changes with *T/W*. For a single vegetation element, the morphology of wind acceleration zone varies with *T/W* and observation height. The area of wind acceleration zone increases at first but decreases then with observation height. And, the area near surface increases with *T/W* monotonously, while the dimensionless area (the ratio of total wind acceleration area versus basal area of vegetation element) near surface decreases at first but increases then with *T/W*. These findings could well explain the disagreement among previous field observations. However, this piece of work is a theoretical and fundamental study for ideal roughness shape. Investigations on wind acceleration with a specific shape of real shrub could be more conducive in practice for field cases. Besides, comparisons and discussion above suggest two limitations of current study on wind acceleration zone around vegetation element. One is the effect of the vegetation porosity; the other is the flow incident angle (or roughness orientation). Their quantitative impacts on wind acceleration should be investigated in the future. Finally, it is hoped that our findings could be helpful to improve our understanding of aeolian transport in sparsely vegetated land in arid and semi-arid region, and wind dispersals of seeds and pollens from shrub vegetation.

## Supplementary information

Supplementary Information

## Data Availability

All data generated or analyzed during this study are included in this published article (and its Supplementary Information Files).

## References

[1] Shao, Y. *Physics and modelling of wind erosion*. Vol. 37 (Springer, 2008).

[2] Kok JF, Parteli EJ, Michaels TI, Karam DB (2012). The physics of wind-blown sand and dust. Rep. Prog. Phys..

[3] Mayaud JR, Webb NP (2017). Vegetation in Drylands: Effects on Wind Flow and Aeolian Sediment Transport. Land.

[4] De Langre E (2008). Effects of wind on plants. Annu. Rev. Fluid Mech..

[5] Virot E, Ponomarenko A, Dehandschoewercker É, Quéré D, Clanet C (2016). Critical wind speed at which trees break. Phys. Rev. E.

[6] Nathan R (2011). Mechanistic models of seed dispersal by wind. Theor. Ecol..

[7] Teller BJ, Zhang R, Shea K (2016). Seed release in a changing climate: initiation of movement increases spread of an invasive species under simulated climate warming. Divers. Distrib..

[8] Raupach M (1992). Drag and drag partition on rough surfaces. Bound.-Lay. Meteorol..

[9] Raupach MR, Gillette DA, Leys JF (1993). The effect of roughness elements on wind erosion threshold. J. Geophys. Res..

[10] Crawley DM, Nickling WG (2003). Drag partition for regularly-arrayed rough surfaces. Bound.-Lay. Meteorol..

[11] Gillies JA, Nickling WG, King J (2007). Shear stress partitioning in large patches of roughness in the atmospheric inertial sublayer. Bound.-Lay. Meteorol..

[12] Brown, S., Nickling, W. G. & Gillies, J. A. A wind tunnel examination of shear stress partitioning for an assortment of surface roughness distributions. *J. Geophys. Res.-Earth*, **113**(F2), 10.1029/2007JF000790 (2008).

[13] Luo W, Lu J, Qian G, Dong Z (2016). Influence of the gap ratio on variations in the surface shear stress and on sand accumulation in the lee of two side-by-side obstacles. Environ. Earth Sci..

[14] Walter B, Gromke C, Leonard KC, Manes C, Lehning M (2012). Spatio-temporal surface shear-stress variability in live plant canopies and cube arrays. Bound.-Lay. Meteorol..

[15] Kang L (2018). Experimental Investigation on Shear-Stress Partitioning for Flexible Plants with Approximately Zero Basal-to-Frontal Area Ratio in a Wind Tunnel. Bound.-Lay. Meteorol..

[16] Kang Liqiang, Zhang Junjie, Zou Xueyong, Cheng Hong, Zhang Chunlai, Yang Zhicheng (2019). Experimental Investigation of the Aerodynamic Roughness Length for Flexible Plants. Boundary-Layer Meteorology.

[17] Xu B, Zhang J, Huang N, Gong K, Liu Y (2018). Characteristics of Turbulent Aeolian Sand Movement Over Straw Checkerboard Barriers and Formation Mechanisms of Their Internal Erosion Form. J. Geophys. Res.-Atmos..

[18] Kang JY, Yoon SC, Shao Y, Kim SW (2011). Comparison of vertical dust flux by implementing three dust emission schemes in WRF/Chem. J. Geophys. Res..

[19] Webb NP, McGowan HA (2009). Approaches to modelling land erodibility by wind. Prog. Phys. Geog..

[20] Webb NP, Okin GS, Brown S (2014). The effect of roughness elements on wind erosion: The importance of surface shear stress distribution. J. Geophys. Res..

[21] Fu LT (2019). Comparisons suggest more efforts are required to parameterize wind flow around shrub vegetation elements for predicting aeolian flux. Sci. Rep..

[22] Okin GS (2008). A new model of wind erosion in the presence of vegetation. J. Geophys. Res..

[23] Leenders JK, Van Boxel JH, Sterk G (2007). The effect of single vegetation elements on wind speed and sediment transport in the Sahelian zone of Burkina Faso. Earth Surf. Proc. Land..

[24] Leenders JK, Sterk G, Van Boxel JH (2011). Modelling wind‐blown sediment transport around single vegetation elements. Earth Surf. Proc. Land..

[25] Mayaud JR, Wiggs GF, Bailey RM (2017). A field‐based parameterization of wind flow recovery in the lee of dryland plants. Earth Surf. Proc. Land..

[26] Ash JE, Wasson RJ (1983). Vegetation and sand mobility in the Australian desert dunefield. Zeitschrift fur Geomorphologie.

[27] Fearnehough W, Fullen MA, Mitchell DJ, Trueman IC, Zhang J (1998). Aeolian deposition and its effect on soil and vegetation changes on stabilised desert dunes in northern China. Geomorphology.

[28] Chopping M (2008). Remote sensing of woody shrub cover in desert grasslands using MISR with a geometric-optical canopy reflectance model. Remote Sens. Environ..

[29] Jiapaer G, Chen X, Bao A (2011). A comparison of methods for estimating fractional vegetation cover in arid regions. Agr. Forest Meteorol..

[30] Cheng H (2018). Wind tunnel study of airflow recovery on the lee side of single plants. Agr. Forest Meteorol..

[31] Cheng H (2019). Transition model for airflow fields from single plants to multiple plants. Agr. Forest Meteorol..

[32] Liu, C., Zheng, Z., Cheng, H. & Zou, X. Airflow around single and multiple plants. *Agr. Forest. Meteorol*. **252**, 27–38, j.agrformet.2018.01.009 (2018).

[33] Bradley EF, Mulhearn PJ (1983). Development of velocity and shear stress distributions in the wake of a porous shelter fence. J. Wind Eng. Indust. Aerodyn..

[34] Sutton, S. L. & McKenna-Neuman, C. Variation in bed level shear stress on surfaces sheltered by nonerodible roughness elements. *J. Geophys. Res.-Earth*, **113**(F3), 10.1029/2007JF000967 (2008).

[35] Walter B, Gromke C, Leonard K, Clifton A, Lehning M (2012). Spatially resolved skin friction velocity measurements using Irwin sensors: a calibration and accuracy analysis. J. Wind Eng. Ind. Aerod..

[36] He W, Huang N, Xu B, Wang W (2018). Numerical simulation of wind-sand movement in the reversed flow region of a sand dune with a bridge built downstream. Eur. Phys. J. E.

[37] Lauder, B. E. & Spalding, D. B. *Lectures in mathematical models of turbulence*. (Academic Press, 1972).

[38] Launder BE, Spalding DB (1974). The numerical computation of turbulent flows. Comput. Method Appl. M..

[39] Liu B, Qu J, Zhang W, Tan L, Gao Y (2014). Numerical evaluation of the scale problem on the wind flow of a windbreak. Sci. Rep..

[40] Cheng NS, Chiew YM (1998). Modified logarithmic law for velocity distribution subjected to upward seepage. J. Hydraul. Eng..

[41] Bouras I, Ma L, Ingham D, Pourkashanian M (2018). An improved k–ω turbulence model for the simulations of the wind turbine wakes in a neutral atmospheric boundary layer flow. J. Wind Eng. Ind. Aerod..

[42] Lima IA, Araújo AD, Parteli EJ, Andrade JS, Herrmann HJ (2017). Optimal array of sand fences. Sci. Rep..

[43] Webb NP, Galloza MS, Zobeck TM, Herrick JE (2016). Threshold wind velocity dynamics as a driver of aeolian sediment mass flux. Aeolian Res..

[44] Sherman DJ (1992). An equilibrium relationship for shear velocity and apparent roughness lenght in aeolian saltation. Geomorphology.

[45] Blocken B, Stathopoulos T, Carmeliet J (2007). CFD simulation of the atmospheric boundary layer: wall function problems. Atmos. Environ..

[46] Kang L, Zhou X, van Hooff T, Blocken B, Gu M (2018). CFD simulation of snow transport over flat, uniformly rough, open terrain: Impact of physical and computational parameters. J. Wind Eng. Ind. Aerod..

[47] Juretić F, Kozmar H (2013). Computational modeling of the neutrally stratified atmospheric boundary layer flow using the standard k–ε turbulence model. J. Wind Eng. Ind. Aerod..

[48] Gillies JA, Nield JM, Nickling WG (2014). Wind speed and sediment transport recovery in the lee of a vegetated and denuded nebkha within a nebkha dune field. Aeolian Res..

[49] Hesp PA, Smyth TA (2017). Nebkha flow dynamics and shadow dune formation. Geomorphology.

[50] Zhang J, Teng Z, Huang N, Guo L, Shao Y (2016). Surface renewal as a significant mechanism for dust emission. Atmos. Chem. Phys..

[51] Gillies JA, Nickling WG, King J (2002). Drag coefficient and plant form response to wind speed in three plant species: Burning Bush (Euonymus alatus), Colorado Blue Spruce (Picea pungens glauca.), and Fountain Grass (Pennisetum setaceum). J. Geophys. Res.-Atmos..

[52] Yang XIA, Meneveau C (2016). Large eddy simulations and parameterisation of roughness element orientation and flow direction effects in rough wall boundary layers. J. Turbul..

[53] Boothroyd RJ, Hardy RJ, Warburton J, Marjoribanks TI (2019). The importance of riparian plant orientation in river flow: implications for flow structures and drag. J. Ecohydraul..

[54] McKenna Neuman C, Langston G (2006). Measurement of water content as a control of particle entrainment by wind. Earth Surf. Proc. Land.: The Journal of the British Geomorphological Research Group.

[55] Haehnel R, Buck N, Song A (2014). Moisture effects on eolian particle entrainment. Environ. Fluid Mech..

[56] Yuge K, Anan M (2019). Evaluation of the Effect of Wind Velocity and Soil Moisture Condition on Soil Erosion in Andosol Agricultural Fields (Model Experiment). Water.

