# Supplementary material for: Wind speed acceleration around a single low solid roughness in atmospheric boundary layer
Source: Sci Rep. 2019 Aug 19;9:12002. doi: 10.1038/s41598-019-48574-7 (PMC6700104; doi:10.1038/s41598-019-48574-7)
Supplement: Supplementary file 1 — Supplementary Information [file 41598_2019_48574_MOESM1_ESM.pdf]

Manuscript

Wind speed acceleration around a single low solid roughness in atmospheric boundary layer

Lin-Tao Fu[1,*,†], Qing Fan[2,†], Zong-Liu Huang[3]

[1]School of Mechanical Engineering, Chengdu University, Chengdu 610106, China

[2]Zhuhai Campus of Zunyi Medical University, Zhuhai 519041, China

[3]Key Laboratory of Fluid and Power Machinery, Ministry of Education, Xihua University, Chengdu 610039, China

[*]Corresponding Email: fultofficial@hotmail.com (L.-T Fu)

Telephone numbers: +86-028-84616075

[†]These authors are contributed equally to this work.

Supplementary information

Figure S1.

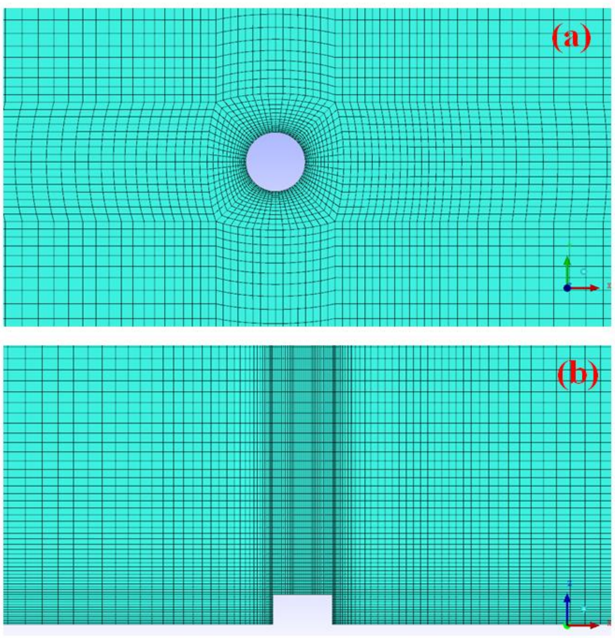

Figure S1. Grid meshes of simulation domain with a single roughness element. Panels (a) and (b) are top view and side view, respectively.

Figure S2.

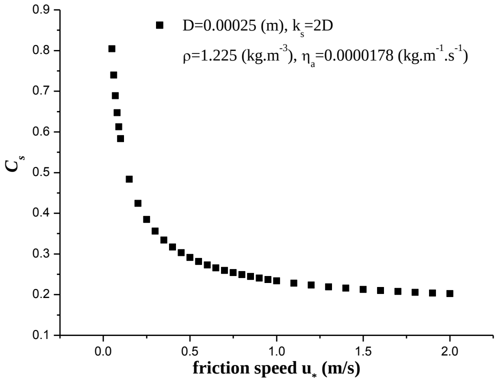

Figure S2. The variation of C_s with friction speed u_* in the case of k_s=2d. The values of C_s are calculated as $C_s = 9.793 z_0 / k_s$, where z_0 is estimated by Equation 6b in main text.

Figure S3.

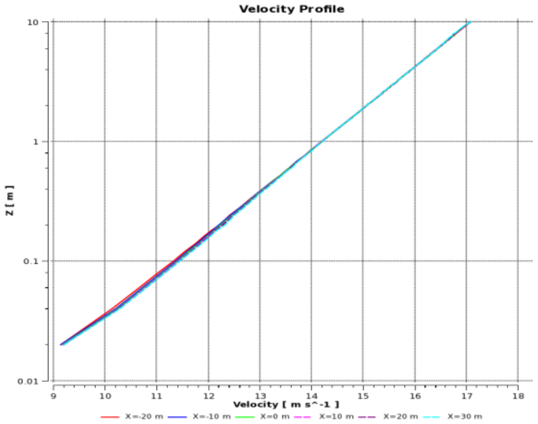

Figure S3. The vertical profiles of time-averaged horizontal speed at different locations from the inlet of simulation domain. In the paper, the origin of coordinate is defined at the center of roughness element, which is 20 meters downwind from the inlet. The *x* coordinate of inlet location is thus -20. The wind data at six locations (x=-20, -10, 0, 10, 20 and 30 m) are shown here.

Figure S4.

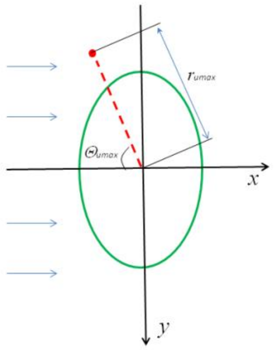

Figure S4. The definitions of r_{umax} and Θ_{umax} in this paper. The dashed red line is the distance (r_{umax}) from the location where the largest wind speed occurs in a horizontal plane to the central location of roughness element. The azimuthal angle (Θ_{umax}) is the angle between the dashed red line and *x* axis.

Figure S5.

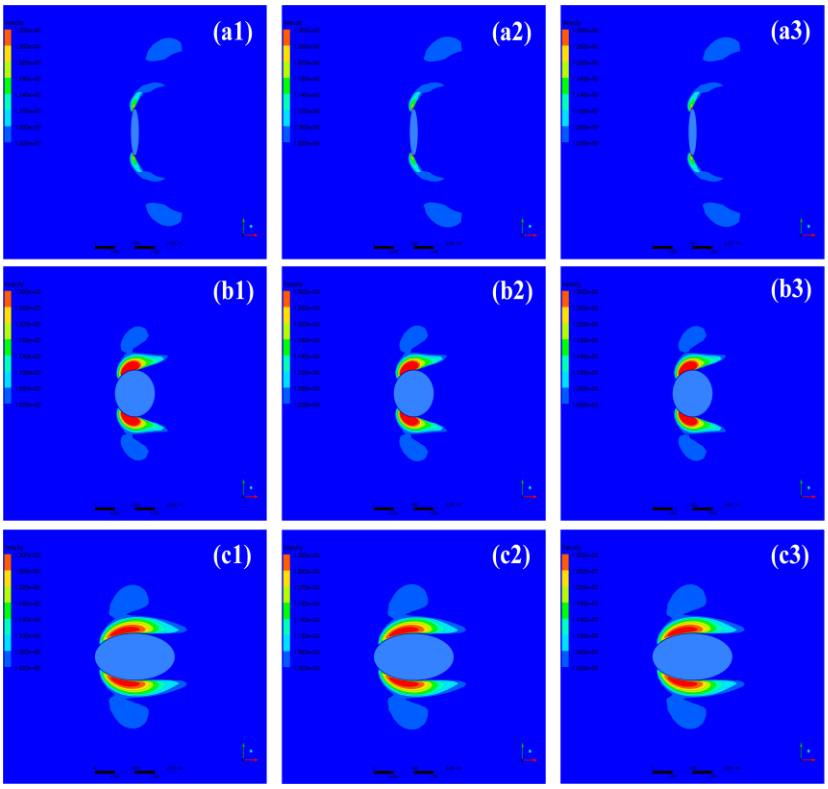

Figure S5. The effects of incoming wind speed on wind acceleration zone at z/H=0.05 for different roughness shapes. Letters a, b and c correspond to T/W =0.2, 1.0 and 2.0, respectively. Numbers 1, 2 and 3 correspond to u_*=0.3, 0.4 and 0.5 m/s, respectively.

Table S1.

Table S1. Statistics on the areas of wind acceleration zone of four friction speeds (u_*=0.25, 0.30, 0.40 and 0.50 m/s) in the case of T/W=0.2. $A_{in,ave}$, $A_{in,min}$, $A_{in,max}$, R_A and P_{RA} are averaged area, minimum area, maximum area, range, and the ratio of range to averaged area, respectively. Here, $R_A=A_{in,max} - A_{in,min}$, $P_{RA}=(A_{in,max} - A_{in,min})/A_{in,ave}$.

z/H	0.05	0.1	0.3	0.5	0.7	0.9	1
$A_{in,ave}$	6.139	28.200	40.802	38.300	34.999	33.326	32.896
$A_{in,min}$	5.408	24.995	38.184	37.167	34.478	33.066	32.713
$A_{in,max}$	6.624	30.238	42.371	38.958	35.354	33.562	33.025
R_A	1.216	5.243	4.187	1.790	0.876	0.496	0.312
P_{RA}	0.198	0.186	0.103	0.047	0.025	0.015	0.009
